# Brain Metastases from Esophageal Squamous Cell Carcinoma: Clinical Characteristics and Prognosis

**DOI:** 10.3389/fonc.2021.652509

**Published:** 2021-04-29

**Authors:** Linlin Xiao, Yvonne M. Mowery, Brian G. Czito, Yajing Wu, Guangbin Gao, Chang Zhai, Jianing Wang, Jun Wang

**Affiliations:** ^1^ Department of Radiation Oncology, Fourth Hospital of Hebei Medical University, Shijiazhuang, China; ^2^ Department of Radiation Oncology, Duke University, Durham, NC, United States

**Keywords:** brain metastases, esophageal squamous cell carcinoma, surgery, brain radiotherapy, GPA score

## Abstract

**Purpose:**

Due to the low incidence of intracranial disease among patients with esophageal cancer (EC), optimal management for these patients has not been established. The aim of this real-world study is to describe the clinical characteristics, treatment approaches, and outcomes for esophageal squamous cell carcinoma (ESCC) patients with brain metastases in order to provide a reference for treatment and associated outcomes of these patients.

**Methods:**

Patients with ESCC treated at the Fourth Hospital of Hebei Medical University between January 1, 2009 and May 31,2020 were identified in an institutional tumor registry. Patients with brain metastases were included for further analysis and categorized by treatment received. Survival was evaluated by the Kaplan-Meier method and Cox proportional hazards models.

**Results:**

Among 19,225 patients with ESCC, 66 (0.34%) were diagnosed with brain metastases. Five patients were treated with surgery, 40 patients were treated with radiotherapy, 10 with systemic therapy alone, and 15 with supportive care alone. The median follow-up time was 7.3 months (95% CI 7.4-11.4). At last follow-up, 59 patients are deceased and 7 patients are alive. Median overall survival (OS) from time of brain metastases diagnosis was 7.6 months (95% CI 5.3-9.9) for all cases. For patients who received locoregional treatment, median OS was 10.9 months (95% CI 7.4-14.3), and survival rates at 6 and 12 months were 75.6% and 37.2%, respectively. For patients without locoregional treatment, median OS was 3.0 months (95% CI 2.5-3.5), and survival rates at 6 and 12 months were 32% and 24%, respectively. OS was significantly improved for patients who received locoregional treatment compared to those treated with systematic treatment alone or supportive care (HR: 2.761, 95% CI 1.509-5.053, P=0.001). The median OS of patients with graded prognostic assessment (GPA) score 0-2 was 6.4 months, compared to median OS of 12.3 months for patients with GPA >2 (HR: 0.507, 95% CI 0.283-0.911).

**Conclusion:**

Brain metastases are rare in patients with ESCC. GPA score maybe a useful prognostic tool for ESCC patients with brain metastases. Receipt of locoregional treatment including brain surgery and radiotherapy was associated with improved survival.

## Introduction

Esophageal cancer (EC) is one of the most common tumors worldwide. In China, 70% of newly diagnosed patients with EC have unresectable or metastatic disease at the time of diagnosis, with spread typically to the liver, bone, lungs and adrenal glands. Brain metastasis remains a rare occurrence with reported rates ranging from 0.3 to 3.8% (1–6). The incidence among patients with esophageal adenocarcinomas (EAC) ranges from 2.0-12.1% (2, 4, 5), which is higher than 0.3-1.4% of patients with esophageal squamous cell carcinoma (ESCC) (2, 4, 5, 7). Prognosis for patients with ESCC and brain metastases is poor, with reported median OS of < 6 months (2–5, 8).

Several studies have demonstrated that surgery or surgery followed by radiotherapy prolongs the survival of patients with brain metastases from EC or other malignancies (3–5, 9). Brain radiotherapy was also an important locoregional therapy for intracranial metastases (10). Given the rare incidence of brain metastases from EC, optimal management for these patients has not been established, and few publications have examined the value of brain radiotherapy in this particular setting as the limitation of data. In this real-world study, we retrospectively reviewed ESCC patients with brain metastases at our institution over the last ten years, evaluating patient clinical characteristics, treatment modalities, possible prognostic factors, and outcomes in order to supply more references for such a rare group of patients.

## Materials and Methods

### Patient Population

For this retrospective cohort study, consecutive patients with EC treated at the Fourth Hospital of Hebei Medical University between January 1, 2009 and May 31, 2020 were identified in an institutional tumor registry through a protocol approved by the institutional review board with waiver of informed consent. In this study, we analyzed the subset of patients with ESCC. All included patients had no history of other malignant tumors, and diagnosis was pathologically confirmed as ESCC. The primary tumor in esophagus was restaged according to the 8^th^ edition of American Joint Committee on Cancer (AJCC) TNM staging classification for carcinoma of the esophagus and esophagogastric junction (8). Brain metastases were diagnosed by contrast-enhanced CT or MRI scans. Graded prognostic assessment (GPA, utilizing age, KPS score, and number of central nervous system and extracranial metastases) was used to estimate the prognosis (9). Brain radiation therapy was administered as stereotactic radiosurgery (SRS) by Gamma Knife, or whole or partial brain radiation by a 6-MV linear accelerator with three-dimensional conformal radiotherapy (3D-CRT) or intensity modulated radiation therapy (IMRT) techniques. All patients were followed through November 30, 2020 by outpatient clinical visit and/or telephone.

### Statistical Analysis

Outcome data was analyzed by SPSS 21.0 statistical software. Overall survival (OS) was defined as the time from the diagnosis of brain metastases until death or last follow-up, with patients censored at date of last follow-up. The efficacy of brain metastases was evaluated according to (response evaluation criteria in solid tumors) RECIST version 1.1. Progression free survival (PFS) was defined as the time from the diagnosis and anti-tumor treatment of brain metastases until disease progression or death or last follow-up, with patients censored at date of last follow-up. The chi squared testing was used for the patient characteristics table. The Kaplan-Meier method was used to estimate OS, and curves were compared by log-rank test. Cox proportional hazard regression analysis was used to perform the univariate and multivariate analysis. All statistical tests were two-sided with p=0.05.

## Results

### Patient Characteristics

During the study period, 19,225 patients with ESCC, 489 patients with EAC, and 275 patients with esophageal small cell carcinoma were evaluated at the Fourth Hospital of Hebei Medical University. Brain metastases were diagnosed in 66 (0.34%) patients with ESCC, 4 (0.82%) patients with EAC and 11 (4%) patients with small cell carcinoma. The current study focuses on the cohort of 66 patients with ESCC and brain metastases, including 50 males and 16 females. Patient characteristics are summarized in **Table 1**. The median patient age at the time of ESCC diagnosis was 63 years (range, 46-83 years), with the majority (63.6%) younger than 65. Most patients had advanced stage at initial diagnosis, including 33 patients with stage III disease (50%) and 24 patients with stage IV disease (36.4%). There were 26 patients with surgery, 26 with radio(chemo)therapy, 12 with chemotherapy and 2 with supportive care alone, for the primary esophagus site at initial diagnosis. Tumors were most commonly located in the mid- (53%) or lower thoracic esophagus (34.8%). The median esophageal tumor length was 6.0 cm (range, 2-12 cm). Thirty-nine patients had a single brain metastasis (59.1%), while 27 patients had multiple brain metastases (40.9%). A total of 31 patients had synchronous extracranial metastases (47%), including in the lung, liver, bone or multiple sites.

**Table 1:** Characteristics of patients with or without locoregional treatment.

Characteristics	Patients with Locoregional Treatment	Patients without Locoregional Treatment	χ^2^ Value	P Value
Gender			1.489	0.222
Male	29	21		
Female	12	4		
Age			0.23	0.632
≤65	27	15		
>65	14	10		
KPS score			0.17	0.68
70-100	31	20		
<70	10	5		
DS-GPA score ^a^			0.001	0.980
0-2	28	17		
>2	13	8		
Group stage at diagnosis			5.3	0.071
II	4	5		
III	25	8		
I V	12	12		
Brain metastases number			0.159	0.690
Single	25	14		
Multiple	16	11		
Extracranial metastases			0.142	0.706
Yes	10	11		
No	21	14		

^a,^DS-GPA Score, Diagnosis-specific graded prognostic assessment.

### Treatment for Brain Metastases

**Figure 1:**
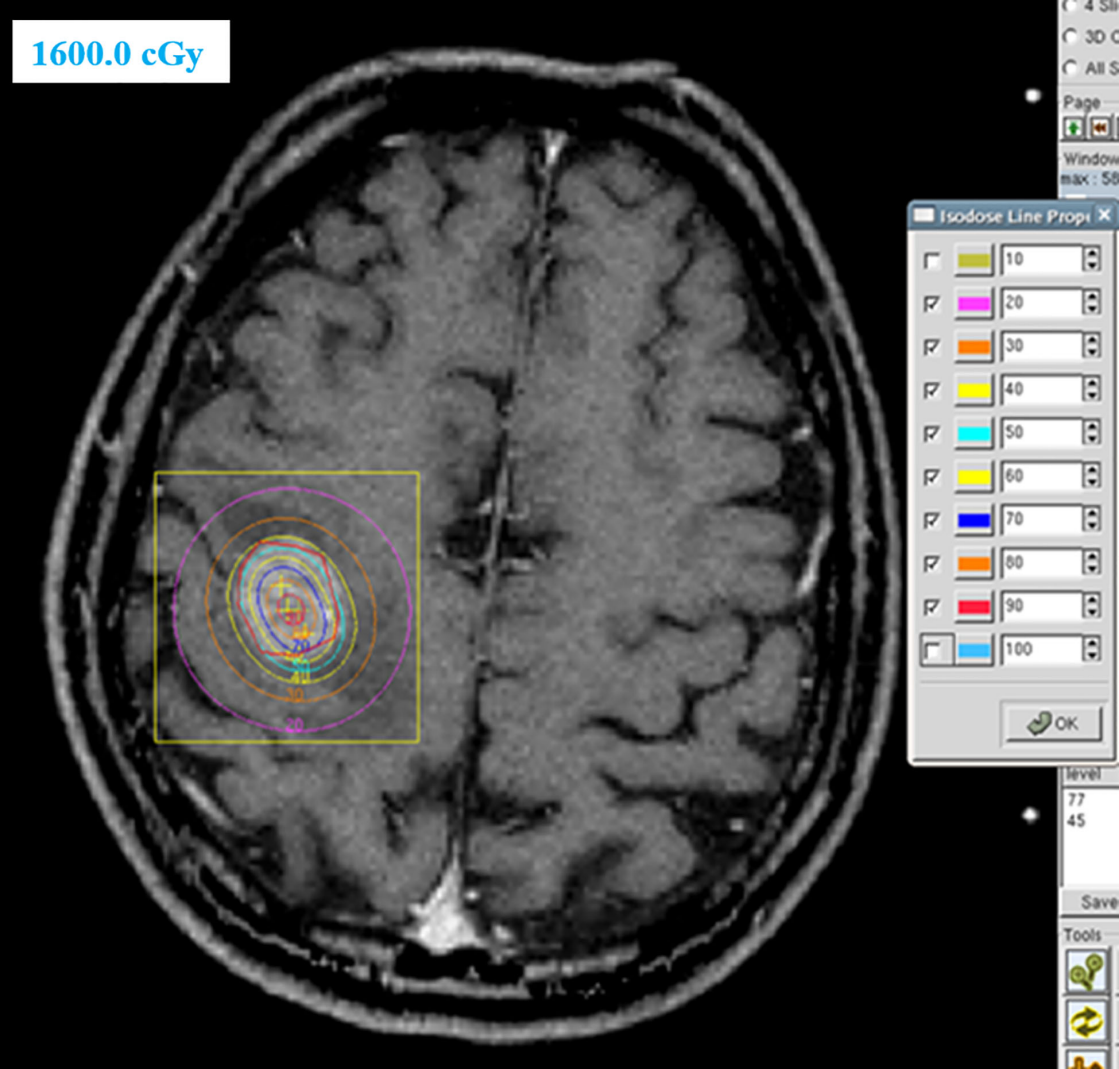
Patient with single metastasis received stereotactic radiosurgery.

**Figure 2:**
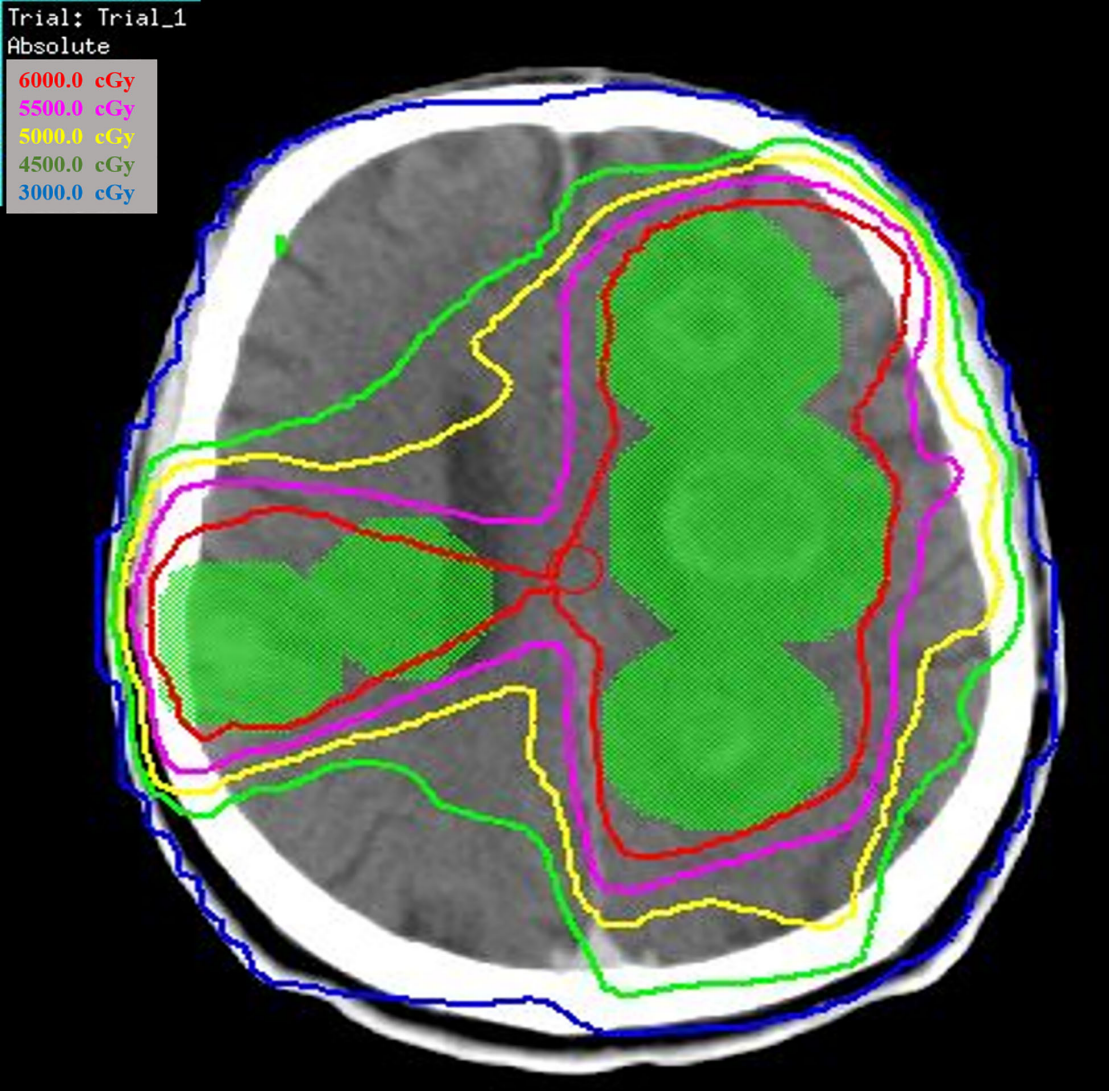
Patient with multiple metastases received conventionally fractionated radiotherapy to the lesions and whole brain radiotherapy.

Of the 66 patients with brain metastases, 41 patients were treated with locoregional treatment, 10 with systemic therapy alone, and 15 with supportive care alone. In the 41 patients treated with locoregional treatment, there were 5 patients with brain surgery (1 patient with brain surgery alone, 4 patients with surgery and radio(chemo)therapy), 40 patients were treated with brain radiotherapy. Brain surgery and radiotherapy was offered based on performance status, nutritional status, controlled extracranial disease and expected survival. Four patient received SRS, 4 patients received SRT (stereotactic radiotherapy) of 50Gy in 10 fractions and 32 patients received conventionally fractionated radiotherapy to the lesion(s) and (or) whole brain radiotherapy. **Figure 1** showed a patient with single metastasis who received SRS. **Figure 2** shows an example of a patient with multiple metastases who received conventionally fractionated radiotherapy to the lesions and whole brain radiotherapy.

Of the 40 patients who received brain radiotherapy, 18 patients received radiotherapy alone, 4 received surgery followed by radio(chemo)therapy, 18 received radiotherapy combined chemotherapy and/or targeted therapy. The median number of cycles of chemotherapy and/or targeted therapy was 2 (range, 1-10).

Of the 10 patients who received systematic treatment alone, 9 patients received chemotherapy with 2-4 cycles of platinum-based docetaxel/paclitaxel, 1 patient received 16 cycles of immune checkpoint inhibitors (ICI).

### Survival

The median follow-up time was 7.3 months (95% CI 7.4-11.4). At last follow-up, 59 patients are deceased and 7 patients are alive. The median interval time from the date of diagnosis of the primary tumor until the date of diagnosis of brain metastases was 9.62 months (range, 0-249.2).

Among the 51 patients who received locoregional treatment and/or systemic therapy, 3 patients achieved complete response (CR) in the brain metastatic sites, 17 patients achieved partial response (PR), 28 patients achieved stable disease (SD) and 3 patients achieved progressive disease (PD) (**Table 2**). For patients with locoregional treatment, there were 2/41 (4.88%) patients with CR, 17/41 (41.46%) patients with PR and 22/41 (53.66%) patients with SD. For patients without locoregional treatment, there were 1/10 (10%) patients with CR, 6/10 (60%) patients with SD and 3/10 (30%) patients with PD.

**Table 2:** The local control of brain metastatic sites in patients with and without locoregional treatment.

Local Control	With Locoregional Treatment	Without Locoregional Treatment
CR	2	1
PR	17	0
SD	22	6
PD	0	3

CR, complete response; PR, partial response; SD, stable disease; PD, progressive disease.

Median OS from time of brain metastases diagnosis was 7.6 months (95% CI 5.3-9.9) for all cases, and survival rates at 6 and 12 months were 59.1% and 32.5%, respectively.

**Figure 3:**
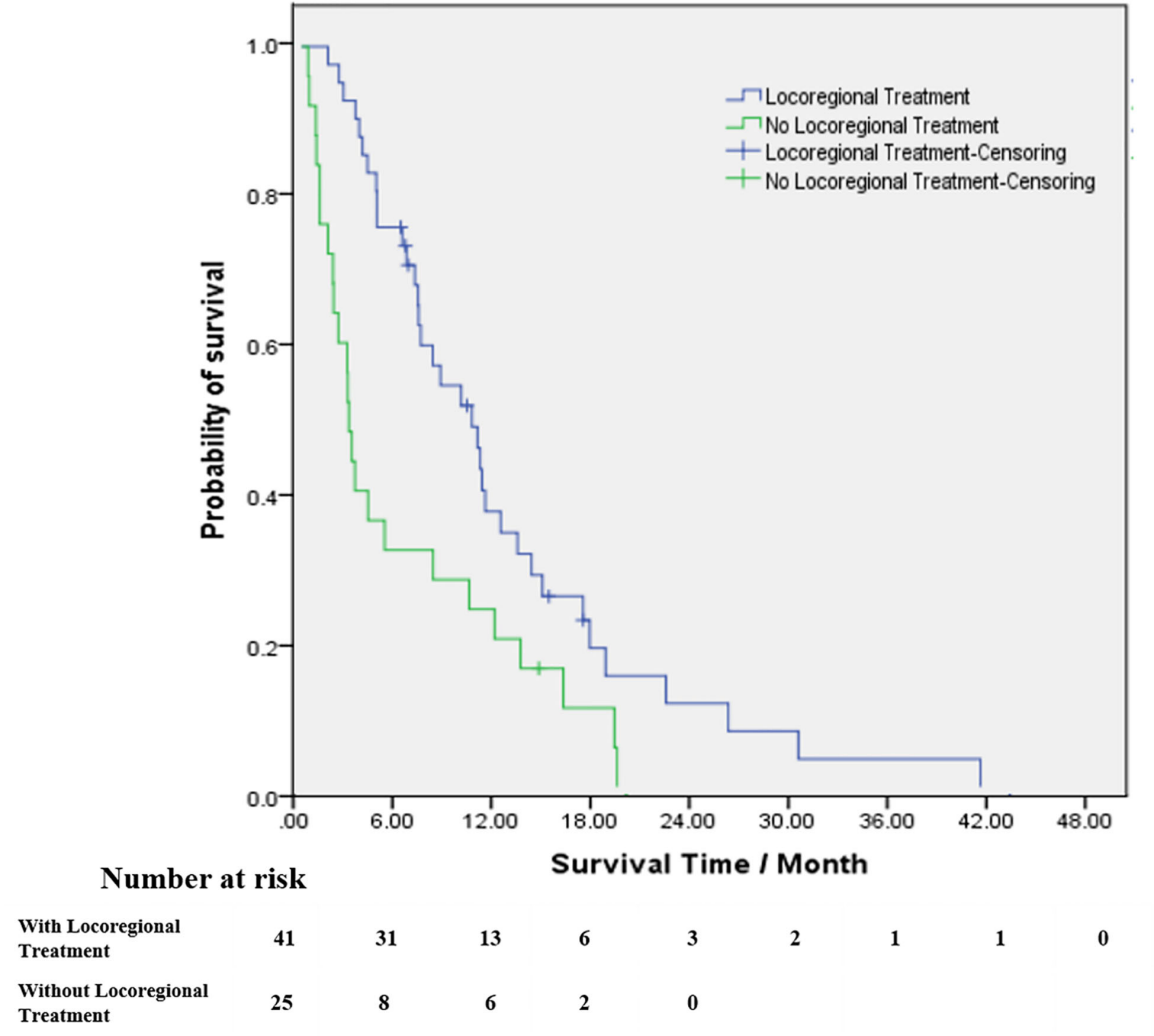
Kaplan–Meier estimates of survival from the time of diagnosis of brain metastases among patients with brain radiotherapy (blue line) and patients without brain radiotherapy (green line).

For patients who received locoregional treatment, median OS was 10.9 months (95% CI 7.4-14.3), and survival rates at 6 and 12 months were 75.6% and 37.2%, respectively. For patients without locoregional treatment, median OS was 3.0 months (95% CI 2.5-3.5), and survival rates at 6 and 12 months were 32% and 24%, respectively. OS was significantly improved for patients who received locoregional treatment compared to those treated with systematic treatment alone or supportive care (HR: 0.471, 95% CI 0.276-0.805, **Table 3** and **Figure 3**). Multivariate analysis demonstrated that locoregional treatment was significantly associated with improved OS (HR: 2.761, 95% CI 1.509-5.053, P=0.001). In addition, for the five patients who received surgery, the median OS was 13.8 months (range, 7.4-18.0). For the four patients who received SRS, the median OS was 10.6 months (range, 6.7-18.0). For the four patients who received SRT, the median OS was 7.2 months (range, 6.6-11.7).

**Table 3:** Univariate analysis of various potential prognostic factors for survival in patients.

Characteristics	Patients Number	Median Survival (Month)	HR	95% CI	P Value
Gender			1.212	0.664-2.233	0.524
Male	50	8.3			
Female	16	7.2			
Age			1.117	0.645-1.934	0.693
≤65	42	8.4			
>65	24	5.3			
KPS score			0.638	0.344-1.182	0.153
<70	15	4.2			
70-100	51	8.4			
GPA Score^a^			0.507	0.283-0.911	**0.023**
0-2	45	6.4			
>2	21	12.3			
Group Stage at Initial Diagnosis			1.272	0.852-1.898	0.240
II	9	11.5			
III	33	7.5			
IV	24	5.3			
Treatment for Primary Site at Initial Diagnosis			1.642	0.605-4.455	0.330
Surgery	26	8.4			
Radio(chemo)therapy	26	14.0			
Chemotherapy	12	2.3			
Supportive care alone	2	3.0			
Brain Metastases Number			0.722	0.424-1.230	0.231
Multiple	27	6.4			
Single	39	8.9			
Extracranial Metastases			0.723	0.427-1.224	0.227
Yes	31	7.2			
No	35	10.9			
Brain Radiotherapy			0.509	0.298-0.870	**0.014**
No	26	3.0			
Yes	40	10.9			
Locoregional treatment			0.471	0.276-0.805	**0.006**
No	24	3.0			
Yes	42	10.9			

^a^GPA Score, graded prognostic assessment.

The bold values means statistically significant.

There were 45 patients with GPA score 0-2 and 21 patients with GPA score >2. The median OS of patients with GPA score 0-2 was 6.4 months, compared to median OS of 12.3 months for patients with GPA >2. OS was significantly improved for patients with high GPA score compared to those with low score (HR: 0.507, 95% CI 0.283-0.911).

## Discussion

Brain metastases from ESCC are relatively rare, as demonstrated by only 0.34% of 19,225 ESCC patients in the current registry being diagnosed with intracranial disease. To the best of our knowledge, this series represents the largest cohort of patients with ESCC and brain metastases so far. As the incidence of brain metastases in patients with EC is low, brain CT or MRI were not routinely performed in these patients. Brain metastases were typically detected when patients exhibited symptoms, and therefore intracranial lesions were typically large at the time of diagnosis.

As an important treatment means, surgery is more suitable for patients with solitary and surgically accessible brain metastases, notably when the systemic disease is controlled and performance status good. Li et al. (7) reported that a median progression-free survival and OS were 14.4 months and 30.1 months, respectively, in 15 ESCC patients with brain metastases. In their study, 92% of patients underwent surgery or SRS. In a series of 22 patients with EC and brain metastases, Welch et al. (5) reported that patients with surgical resection plus radiotherapy exhibited better OS compared to patients with brain radiotherapy alone (median OS: 13.5 vs. 3 months, P=0.003). Yoshida (9) analyzed the outcomes of 17 patients with brain metastases from EC and demonstrated that the median survival of 7 patients after resection alone was 17.7 months compared to 65.5 months in the 3 patients treated with resection plus radiation.* *While these studies are limited by their retrospective nature and potential confounding factors such as improved performance status correlating with surgical intervention, these data suggest that, where feasible, neurosurgical resection followed by radiotherapy is likely the best treatment approach for these patients. There were five patients with surgery in the current study, including 1 patient with surgery followed by chemotherapy, 2 patients with surgery followed by radiotherapy and 2 patents with surgery followed by chemoradiotherapy. The median OS was 13.8 months (range, 7.4-18.0). While as the limitation of the data, we could not compare the prognosis among patients with brain surgery, surgery followed by radiotherapy and brain radiotherapy with or without systematic treatment.

Brain radiotherapy was another standard locoregional therapy for intracranial metastases. Given the rare incidence of brain metastases from EC, few publications have examined the value of brain radiotherapy in this particular setting. In this study, brain radiotherapy was administered to 40 patients. Median OS was 10.9 months for patients receiving brain radiotherapy, which was significantly longer than the 3.0 months of patients without brain radiotherapy (HR: 0.509, 95% CI 0.298-0.870 P=0.014).

Brain had been viewed as immunologically isolated from the peripheral immune system, which was known as “immunologically privileged”. While recent literatures have shown that SRS could promote the release of some antigens in central nervous system and counteract some immunosuppressive processes, then treating patients with brain metastases by the potential synergy between radiation and ICI (13–16). A meta-analysis included 17 studies, involving 534 patients with brain metastases and treated with SRS/ICI (17). The results demonstrated that ICI combined with SRS could significantly improve the 1-year OS (64.6% vs. 51.6%, P=0.00027) compared with SRS alone. Chen et al. also found that ICI combined with SRS may decrease the incidence of new brain metastases and bring favorable survival outcomes without increased rates of adverse events (18). Recently, the KEYNOTE-181, ATTRACTION-3 and ESCORT study had demonstrated that ICI was associated with a significant improvement in OS and a manageable toxicity profile compared with chemotherapy in previously treated patients with advanced or metastatic EC, representing a standard second-line treatment option for these patients (19–21). While the three studies did not include EC patients with brain metastases, so the effect of ICI was not sure for these patients. More studies were expected to explore the effect of ICI combined with SRS in patients with brain metastases more than melanoma and non-small cell lung cancer. In this study, one patient received sixteen cycles of ICI and he was still alive for 16 months from he was diagnosed with brain metastases in July 26, 2019. And the last check-up showed that the lesion in the brain had been lost. The efficacy of ICI in patients with brain metastases needs more exploration and validation.

Due to its quantitative nature, the GPA score is an objective prognostic index used to estimate expected OS (9). Li et al. (7) demonstrated that patients with a GPA score of 0-2.0 achieved median OS of 4.6 months compared to 31.5 months for patients with GPA scores 2.5-3.0 (P<0.01). In the current study, median OS of patients with GPA score of 0-2.0 versus >2.0 was 6.4 months versus 12.3 months, respectively (HR: 0.507, 95% CI 0.283-0.911, P=0.023). GPA score may be a useful prognostic tool for ESCC patients with brain metastases. Given their improved prognosis, locoregional treatment should be considered for patients with a GPA score over 2.0.

Presenting stage has also been associated with the occurrence of brain metastases in EC. Weinberg et al. (2) demonstrated that 70% of patients had extracerebral distant lesions at the time of diagnosis with intracranial metastases. Similarly, 81% of EC patients with brain metastasis had clinical Stage III–IV tumors in the study by Ogawa et al. (3). In the current study, 86.4% of patients diagnosed with brain metastases were diagnosed with stage III or IV disease at initial presentation, consistent with previous studies (2–5, 22). Late stage at diagnosis and disease progression may be associated with impaired immune function, contributing to the higher incidence of brain metastases. Similarly, Gabrielsen et al. (22) reported that primary esophageal tumor length was associated with the occurrence of brain metastases (mean length: 8.63 cm vs. 5.12 cm, P<0.001). In the Ogawa et al. (3) series of EC patients with brain metastases, the mean primary tumor length was 8.2 cm (range, 2-19 cm). In the current study, the mean length of esophageal tumor was 6.0cm (range, 2-12 cm), which is shorter than in other similar studies. Additional studies are necessary to determine the association between esophageal tumor length and brain metastases development. Takeshima et al. (23) showed that a longer disease-free interval between initial diagnosis and development of intracranial disease has been associated with better prognosis. In the current study, there was no significant difference in survival between the group of patients diagnosed with brain metastases within 6 months versus >6 months after EC diagnosis (median OS 7.5 months vs. 8.4 months, HR 0.785, 95% CI 0.451-1.367). In addition, other studies have demonstrated that OS is not only associated with treatment modalities and disease-free interval, but also with performance status (3, 8, 24). In current study, the median survival of patients with KPS scores of 70-100 was 8.4 months, which was higher than 4.2 months for patients with KPS scores of <70 (HR 0.638, 95% CI 0.344-1.182). Thus, for patients with poor performance status and short expected OS, potentially burdensome overtreatment with brain radiotherapy or surgery may be avoided.

More globally, most patients with brain metastases have either primary lung cancer or metastatic disease involving the lung. However, in patients with EC, brain metastases have not been directly associated with lung metastases. Weinberg et al. (2) reported that 74% of patients with brain metastases did not have lung metastases. Ogawa et al. (3) reported that 69% of patients with brain metastases did not have lung metastases. In the current study, 77.3% of patients with brain metastases did not have lung metastases. Potential theory for the low overlap of brain and lung metastases for EC is that spread of tumor cells to the brain occurs through the Batson venous plexus (2, 3).

There are some limitations to this study. Firstly, it was a retrospective study and the treatment options were heterogeneous, so we could not compare brain surgery with radiotherapy, conventional fractionated brain radiotherapy with SRS or SRT in order to better assess locoregional treatment options. However, our results and those of previous studies indicate that locoregional treatment can significantly improve the survival of patients with brain metastases. Secondly, brain imaging is not routinely performed for this patient population, so some asymptomatic brain metastases may not have been diagnosed and thus were not included in this series.

In conclusion, the development of symptomatic brain metastases is rare for patients with ESCC. Locoregional treatment is associated with improved OS in our study. Thus, brain surgery and radiation therapy should be considered for patients with brain metastases from ESCC with good performance status. In addition, GPA score may be a useful prognostic tool for ESCC patients with brain metastases. Given their improved prognosis, locoregional treatment should be more considered for patients with a GPA score over 2.0. Given limitations of our study, further study is needed to confirm these findings and compare the efficacy and safety of different locoregional treatment options and explore more effective systematic treatment.

## Data Availability Statement

The raw data supporting the conclusions of this article will be made available by the authors, without undue reservation.

## Ethics Statement

Ethical review and approval was not required for the study on human participants, in accordance with the local legislation and institutional requirements.

## Author Contributions

Conceived and designed the study: JuW. Performed the study and analyzed the data: LX, YW, GG, CZ, JiW. Wrote the paper: LX, YM, BC. Supervised the entire study and review the final paper: JuW, BC, YM. All authors contributed to the article and approved the submitted version.

## Funding

This study was funded by the 2016 Beijing Hope Marathon Fund of China Cancer Foundation (LC2016W10), Medical Science Research Key Projects of Hebei Provincial Department (ZD20140060), and Hebei Clinical Research Center for Radiation Oncology and Oncology Department of Hebei Medical University.

## Conflict of Interest

The authors declare that the research was conducted in the absence of any commercial or financial relationships that could be construed as a potential conflict of interest.
