# Corrigendum: Brain Metastases from Esophageal Squamous Cell Carcinoma: Clinical Characteristics and Prognosis

**DOI:** 10.3389/fonc.2022.827810

**Published:** 2022-02-04

**Authors:** Linlin Xiao, Yvonne M. Mowery, Brian G. Czito, Yajing Wu, Guangbin Gao, Chang Zhai, Jianing Wang, Jun Wang

**Affiliations:** ^1^ Department of Radiation Oncology, Fourth Hospital of Hebei Medical University, Shijiazhuang, China; ^2^ Department of Radiation Oncology, Duke University, Durham, NC, United States

**Keywords:** brain metastases, esophageal squamous cell carcinoma, surgery, brain radiotherapy, GPA score

In the original article, there was a mistake in **Table 3** as published. We made the mistake of writing “GPA” as “DS-GPA”. The corrected **Table 3** appears below.

**Table 3:** Univariate analysis of various potential prognostic factors for survival in patients.

Characteristics	Patients Number	Median Survival (Month)	HR	95% CI	P Value
Gender			1.212	0.664-2.233	0.524
Male	50	8.3			
Female	16	7.2			
Age			1.117	0.645-1.934	0.693
≤65	42	8.4			
>65	24	5.3			
KPS score			0.638	0.344-1.182	0.153
<70	15	4.2			
70-100	51	8.4			
GPA Score^a^			0.507	0.283-0.911	**0.023**
0-2	45	6.4			
>2	21	12.3			
Group Stage at Initial Diagnosis			1.272	0.852-1.898	0.240
II	9	11.5			
III	33	7.5			
IV	24	5.3			
Treatment for Primary Site at Initial Diagnosis			1.642	0.605-4.455	0.330
Surgery	26	8.4			
Radio(chemo)therapy	26	14.0			
Chemotherapy	12	2.3			
Supportive care alone	2	3.0			
Brain Metastases Number			0.722	0.424-1.230	0.231
Multiple	27	6.4			
Single	39	8.9			
Extracranial Metastases			0.723	0.427-1.224	0.227
Yes	31	7.2			
No	35	10.9			
Brain Radiotherapy			0.509	0.298-0.870	**0.014**
No	26	3.0			
Yes	40	10.9			
Locoregional treatment			0.471	0.276-0.805	**0.006**
No	24	3.0			
Yes	42	10.9			

^a^GPA Score, graded prognostic assessment.

The bold values means statistically significant.

In the original article, there was an error. We made the mistake of writing “GPA” as “DS-GPA”. A correction has been made to the Abstract. The corrected section appears below.

Among 19,225 patients with ESCC, 66 (0.34%) were diagnosed with brain metastases. Five patients were treated with surgery, 40 patients were treated with radiotherapy, 10 with systemic therapy alone, and 15 with supportive care alone. The median follow-up time was 7.3 months (95% CI 7.4-11.4). At last follow-up, 59 patients are deceased and 7 patients are alive. Median overall survival (OS) from time of brain metastases diagnosis was 7.6 months (95% CI 5.3-9.9) for all cases. For patients who received locoregional treatment, median OS was 10.9 months (95% CI 7.4-14.3), and survival rates at 6 and 12 months were 75.6% and 37.2%, respectively. For patients without locoregional treatment, median OS was 3.0 months (95% CI 2.5-3.5), and survival rates at 6 and 12 months were 32% and 24%, respectively. OS was significantly improved for patients who received locoregional treatment compared to those treated with systematic treatment alone or supportive care (HR: 2.761, 95% CI 1.509-5.053, P=0.001). The median OS of patients with graded prognostic assessment (GPA) score 0-2 was 6.4 months, compared to median OS of 12.3 months for patients with GPA >2 (HR: 0.507, 95% CI 0.283-0.911).

A correction has been made to the *Conclusion*. The corrected section appears below.

Brain metastases are rare in patients with ESCC. GPA score maybe a useful prognostic tool for ESCC patients with brain metastases. Receipt of locoregional treatment including brain surgery and radiotherapy was associated with improved survival.

A correction has been made to the keywords. The corrected section appears below.

brain metastases, esophageal squamous cell carcinoma, surgery, brain radiotherapy, GPA score.

A correction has been made to *Materials and Methods*, *Patient Population.* The corrected section appears below.

For this retrospective cohort study, consecutive patients with EC treated at the Fourth Hospital of Hebei Medical University between January 1, 2009 and May 31, 2020 were identified in an institutional tumor registry through a protocol approved by the institutional review board with waiver of informed consent. In this study, we analyzed the subset of patients with ESCC. All included patients had no history of other malignant tumors, and diagnosis was pathologically confirmed as ESCC. The primary tumor in esophagus was restaged according to the 8^th^ edition of American Joint Committee on Cancer (AJCC) TNM staging classification for carcinoma of the esophagus and esophagogastric junction (8). Brain metastases were diagnosed by contrast-enhanced CT or MRI scans. Graded prognostic assessment (GPA, utilizing age, KPS score, and number of central nervous system and extracranial metastases) was used to estimate the prognosis (9). Brain radiation therapy was administered as stereotactic radiosurgery (SRS) by Gamma Knife, or whole or partial brain radiation by a 6-MV linear accelerator with three-dimensional conformal radiotherapy (3D-CRT) or intensity modulated radiation therapy (IMRT) techniques. All patients were followed through November 30, 2020 by outpatient clinical visit and/or telephone.

A correction has been made to *Results*, *Survival, Paragraph 5.* The corrected paragraph appears below.

There were 45 patients with GPA score 0-2 and 21 patients with GPA score >2. The median OS of patients with GPA score 0-2 was 6.4 months, compared to median OS of 12.3 months for patients with GPA >2. OS was significantly improved for patients with high GPA score compared to those with low score (HR: 0.507, 95% CI 0.283-0.911).

A correction has been made to *Discussion, Paragraph 5.* The corrected paragraph appears below.

Due to its quantitative nature, the GPA score is an objective prognostic index used to estimate expected OS (9). Li et al. (7) demonstrated that patients with a GPA score of 0-2.0 achieved median OS of 4.6 months compared to 31.5 months for patients with GPA scores 2.5-3.0 (P<0.01). In the current study, median OS of patients with GPA score of 0-2.0 versus >2.0 was 6.4 months versus 12.3 months, respectively (HR: 0.507, 95% CI 0.283-0.911, P=0.023). GPA score may be a useful prognostic tool for ESCC patients with brain metastases. Given their improved prognosis, locoregional treatment should be considered for patients with a GPA score over 2.0.

A correction has been made to *Discussion, Paragraph 9.* The corrected paragraph appears below.

In conclusion, the development of symptomatic brain metastases is rare for patients with ESCC. Locoregional treatment is associated with improved OS in our study. Thus, brain surgery and radiation therapy should be considered for patients with brain metastases from ESCC with good performance status. In addition, GPA score may be a useful prognostic tool for ESCC patients with brain metastases. Given their improved prognosis, locoregional treatment should be more considered for patients with a GPA score over 2.0. Given limitations of our study, further study is needed to confirm these findings and compare the efficacy and safety of different locoregional treatment options and explore more effective systematic treatment.

The authors apologize for these errors and state that this does not change the scientific conclusions of the article in any way. The original article has been updated.

## Publisher’s Note

All claims expressed in this article are solely those of the authors and do not necessarily represent those of their affiliated organizations, or those of the publisher, the editors and the reviewers. Any product that may be evaluated in this article, or claim that may be made by its manufacturer, is not guaranteed or endorsed by the publisher.

